# Correlations between clinical and physiological consequences of the novel mutation R878C in a highly conserved pore residue in the cardiac Na^+^ channel

**DOI:** 10.1111/j.1748-1716.2008.01883.x

**Published:** 2008-12

**Authors:** Y Zhang, T Wang, A Ma, X Zhou, J Gui, H Wan, R Shi, C Huang, A A Grace, C L-H Huang, D Trump, H Zhang, T Zimmer, M Lei

**Affiliations:** 1 Cardiovascular Ion Channel Disease Laboratory, Department of Paediatrics, First Affiliated Hospital, Medical College of Xi’an Jiaotong UniversityXi’an, China; 2 Medical Genetics Research Group and Cardiovascular Research Group, School of Clinical and Laboratory Sciences, The University ofManchester, ManchesterUK; 3 Cardiovascular Biology Group, Department of Biochemistry and Physiological Laboratory, University of CambridgeCambridge, UK; 4 Biological Physics Group, School of Physics & Astronomy, The University of ManchesterManchester, UK; 5 Institute of Physiology II, Friedrich Schiller UniversityJena, Germany

**Keywords:** cardiac Na^+^ channels, novel mutation, pore-forming region, *SCN5A*, sick sinus syndrome

## Abstract

**Aim:**

We compared the clinical and physiological consequences of the novel mutation R878C in a highly conserved pore residue in domain II (S5-S6) of human, hNa_v_1.5, cardiac Na^+^ channels.

**Methods:**

Full clinical evaluation of pedigree members through three generations of a Chinese family combined with *SCN5A* sequencing from genomic DNA was compared with patch and voltage-clamp results from two independent expression systems.

**Results:**

The four mutation carriers showed bradycardia, and slowed sino-atrial, atrioventricular and intraventricular conduction. Two also showed sick sinus syndrome; two had ST elevation in leads V1 and V2. Unlike WT-hNa_v_1.5, whole-cell patch-clamped HEK293 cells expressing R878C-hNa_v_1.5 showed no detectable Na^+^ currents (*i*_Na_), even with substitution of a similarly charged lysine residue. Voltage-clamped *Xenopus* oocytes injected with either 0.04 or 1.5 μg μL^−1^ R878C-hNa_v_1.5 cRNA similarly showed no *i*_Na_, yet WT-hNa_v_1.5 cRNA diluted to 0.0004–0.0008 ng μL^−1^resulted in expression of detectable *i*_Na_. *i*_Na_ was simply determined by the amount of injected WT-hNa_v_1.5: doubling the dose of WT-hNa_v_1.5 cRNA doubled *i*_Na_. *i*_Na_ amplitudes and activation and inactivation characteristics were similar irrespective of whether WT-hNa_v_1.5 cRNA was given alone or combined with equal doses of R878C-hNa_v_1.5 cRNA therefore excluding dominant negative phenotypic effects. Na^+^ channel function in HEK293 cells transfected with R878C-hNa_v_1.5 was not restored by exposure to mexiletine (200 μm) and lidocaine (100 μm). Fluorescence confocal microscopy using E3-Nav1.5 antibody demonstrated persistent membrane expression of both WT and R878C-hNa_v_1.5. Modelling studies confirmed that such *i*_Na_ reductions reproduced the SSS phenotype.

**Conclusion:**

Clinical consequences of the novel R878C mutation correlate with results of physiological studies.

Mutations in *SCN5A* encoding the pore-forming α-subunit of the human cardiac Na^+^ channel have been associated with an increasingly wide range of cardiac rhythm disorders that include the long QT syndrome type 3 (LQT3), Brugada syndrome (BrS) and cardiac conduction diseases, idiopathic ventricular fibrillation, sinus node dysfunction including sick sinus syndrome (SSS), atrial standstill and sudden infant death syndrome (SIDS) (Akai *et al.* 2000, Benson *et al.* 2003, Groenewegen *et al.* 2003, Tan *et al.* 2003, Wang *et al.* 2007). Furthermore, recent clinical reports, often associated with physiological studies, suggest that particular mutations in Na^+^ channels result in a wide range of electrophysiological changes as reflected in electrocardiographic (ECG) studies. For example, the gain of function, C-terminal *SCN5A* gene mutation (1795insD) in a large Dutch family resulted in bradycardia, conduction disease, LQT3 and BrS (Bezzina *et al.* 1999, van den Berg *et al.* 2001). Mice carrying its murine equivalent similarly displayed bradycardia, right ventricular conduction slowing and QT prolongation (Remme *et al.* 2006). Conversely, loss of function mutations result in a reduction in total *i*_Na_ (Bennett *et al.* 1995, Chen *et al.* 1998, Tan *et al.* 2003). For example, the E161K mutation in the Na^+^ channel is correspondingly associated with clinical and ECG features of BrS, conduction disease and sinus node dysfunction (Smits *et al.* 2005). Mice with a single null mutation in *Scn5a* correspondingly show reduced Na^+^ channel function and electrophysiological defects. These include impaired atrioventricular conduction, delayed intramyocardial conduction and ventricular tachycardia with characteristics of re-entrant excitation (Papadatos *et al.* 2002).

The present study identifies and characterizes a novel missense mutation of the highly conserved pore residue arginine to cysteine R878C in domain II S5-S6 of the cardiac Na^+^ channel, and associates it with clinical phenotypes that include ECG features of slowed atrioventricular conduction, SSS, and ST elevations in the V1 and V2 leads in three generations of a Chinese family. Our biophysical studies involving mutant channel expression in either HEK293 cells or *Xenopus* oocytes suggested that the above mutation similarly results in non-functional Na^+^ channels, thereby attributing clinical findings of a wide range of phenotypes to a single physiological alteration in Na^+^ channel function. Function was not restored by the substitution R878K involving a similarly positively charged lysine reflecting a critical function for the R878 residue. It was also not restored by transient exposures to mexiletine (200 μm) and lidocaine (100 μm), manoeuvres that have previously been used to restore channel trafficking (Rajamani *et al.* 2002, Valdivia *et al.* 2004, Anderson *et al.* 2006, Cordeiro *et al.* 2006). Furthermore, we found that the amount of measurable current is simply determined by the level of expression of the WT-hNa_v_1.5 channel: R878C-hNa_v_1.*5* did not exert dominant negative phenotypic effects. Immunochemical studies suggested a persistent membrane expression of both WT and mutant Na^+^ channels. Numerical simulations of the effect of the mutant channel on sino-atrial (SA) node function then reproduced the SSS phenotype. This analysis of the R878C mutation thus extends from its clinical manifestations to physiological characterization and modelling.

## Methods

### Clinical investigation

**Figure 1:**
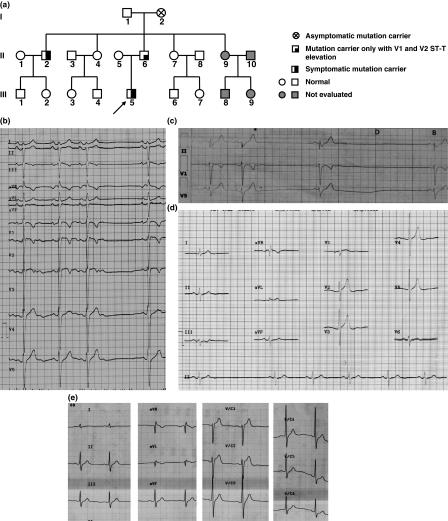
Pedigree and electrocardiographic features of the affected family. (a) Pedigree of the family with phenotypic and genotypic information. Arrow indicates the proband. (b, c) ECG and Holter data of proband. (d) Twelve-lead ECG of II-2; (e) 12-lead ECG of II-6.

All investigations conformed to principles defined in the Helsinki Declaration. Seventeen members of a three-generation Chinese family were investigated (see Fig. 1a), informed written consent having been obtained from each member. They included a full medical history, physical examination, at least two 12-lead ECG recordings obtained at different times and echocardiographic scanning. Heart rates, widths of P wave, PR intervals, QRS durations and QT intervals, corrected for heart rate (QTc), were measured in limb lead II (or lead I or III if it could not be measured exactly with lead II) using Bazett’s formula. QTc intervals were averaged from five consecutive beats of at least two 12-lead ECG recordings at different time points. The presence or absence of ST elevation was assessed using lead V2. Two 24-h Holter-ECG recordings were performed in each of the affected individuals. Two hundred unrelated control individuals were randomly selected from a group of Chinese healthy volunteers with normal 12-lead ECGs and without reported cardiovascular history.

### SCN5A mutation analysis

Genomic DNA was extracted from 10 mL of whole blood using the Puregene Isolation kit (Gentra Systems, Minneapolis, MN, USA). The coding regions of SCN5A and exon/intron boundaries were amplified from genomic DNA by polymerase chain reaction (PCR) using a comprehensive set of primers and PCR conditions as described previously (Wang *et al.* 1995, Syrris *et al.* 2001). Bidirectional sequencing was performed by an ABI automated cycle sequencer using an ABI BigDye Terminator Cycle Sequencing Kit (Applied Biosystems, Foster City, CA, USA). Sequencing results were aligned with the respective wild-type (WT) sequences to detect genetic variations. Identified mutation was screened in the remaining family members by direct DNA sequencing. The mutations were further verified by single strand conformational polymorphism (SSCP) analysis. The same mutation was also screened in 200 controls with the same ethnic background.

### Generation of expressing construct and cRNA encoding mutant human Na_v_1.5

Plasmid pSP64T-hH1 coding for WT human Na_v_1.5 (hNa_v_1.5 or hH1, accession No. M77235, kindly provided by Dr A. L. George, Vanderbilt University, TN, USA) was used as template for generating R878C mutant Na_v_1.5 cDNA by recombinant PCR using the primer pair 5′-AAGGCATGAAAGAAGTCCATCATGTGCCAGCAAGGCAGCAGGCCTGAGTC-3′ (for R878C). The PCR product was then inserted as BseJI/Asp718I (R878C) fragment into the corresponding site of the WT-hNa_v_1.5 clone. The resulting DNA construct was checked by restriction analysis, and nucleotide exchange was confirmed by DNA sequencing. Following *Xba* I digestion of the plasmid, capped cRNA was prepared by *in vitro* transcription reaction with SP6 RNA polymerase (Roche Diagnostics GmbH, Mannheim, Germany). The WT-hNa_v_1.5 was subcloned into a pEGFP expression vector. pEGFP-R878C-hNa_v_1.5 was generated by site-directed mutagenesis (QuickChange^TM^ Site-Directed Mutagenesis Kit; Stratagene, Cedar Creek, TX, USA) using pEGFP-WT-hNa_v_1.5 as the template for transfection of HEK293 cells used in the biophysical studies. Finally, WT-hNa_v_1.5 was subcloned into pEGFP, pEYFP and pECFP expression vector. pEGFP-R878C-hNa_v_1.5 and pEYFP-R878C-hNa_v_1.5 were generated as described above, using pEGFP-WT-hNa_v_1.5 and pEYFP-WT-hNa_v_1.5 as templates, respectively, and used for transfection of HEK293 cells for immunochemical staining visualized by confocal microscopy.

### Expression of human Na_v_1.5 in HEK293 cells and *Xenopus* oocytes

HEK293 cells were cultured and transiently transfected, either in the presence or absence of the plasmid for the β_1_ Na^+^ channel subunit (also provided by Dr A. L. George) using LipofectAMINE (Invitrogen, Carlsbad, CA, USA) as described previously (Wang *et al.* 2002). Oocytes were prepared from *Xenopus laevis* and cRNA injection was performed according to established procedures (Zimmer *et al.* 2002a). We used two independent R878C hNa_v_1.5 clones for electrophysiological measurements. For current comparisons, the different cRNA samples were diluted in order to adjust the cRNA concentration for each variant to ∼0.04 ng μL^−1^. In the case of R878C we also injected undiluted cRNA at ∼1.5 μg μL^−1^. Typical volumes of such injectates were between 40 and 60 nL. After 3 days of incubation at 18 °C in Barth medium, the peak current amplitude of the whole-cell hNa_v_1.5 current was usually between 0.5 and 6.0 μA. Measurements were performed in at least three different batches of oocytes.

### Electrophysiological studies

For electrophysiological recordings, the HEK293 cells were trypsinized 24 h after transfection and seeded onto a glass coverslip to a density that enabled single cells to be identified. Whole-cell Na^+^ currents in HEK293 cells were studied by patch-clamp recordings with patch pipettes fabricated from borosilicate glass capillaries (1.5 mm OD; Fisher Scientific, Pittsburgh, PA, USA). The pipettes were pulled with a gravity puller (model PP-830; Narishige, Tokyo, Japan) and filled with a pipette solution of the following composition (in mm) KCl 10, CsF 105, NaCl 10, HEPES 10, titrated to pH 7.2 with CsOH. Pipette resistance ranged from 1.0 to 2.0 MΩ when pipettes were filled with the internal solution. The perfusion solution contained (in mm): NaCl 130, KCl 5, CaCl_2_ 1.8, MgCl_2_ 1.0, sodium acetate 2.8, HEPES 10, and glucose 10, titrated to pH 7.4 with NaOH. Current signals were recorded with an Axon 200B amplifier (Axon Instruments, Foster City, CA, USA), and series resistance errors were reduced by approximately 70∼80% with electronic compensation. Signals were acquired at 20 kHz (Digidata 1200; Axon Instruments) and analysed with a PC running pclamp 9 software (Axon Instruments). All recordings were made at room temperature (20–22 °C). Whole-cell Na^+^ currents in *Xenopus* oocytes were recorded with the two-microelectrode voltage clamp technique using a commercial amplifier (OC725C; Warner Instruments, Hamden, CT, USA), as previously described (Zimmer *et al.* 2002a). The glass microelectrodes were filled with 3 m KCl. The microelectrode resistance was between 0.2 and 0.5 MΩ. The bath solution contained (in mm): 96 NaCl, 2 KCl, 1.8 CaCl_2_, 10 HEPES titrated to pH 7.2 with KOH. The currents were elicited by test potentials from −80 to 40 mV using a holding potential of −120 mV. The pulsing frequency was 1 Hz. Recording and analysis of the data were performed on a PC with iso2 software (MFK, Niedernhausen, Germany). The sampling rate was generally 20 kHz.

### Immunocytochemistry and imaging confocal microscopy

HEK293 cells expressing GFP-, CFP- and YFP-tagged hNa_v_1.5 were fixed with 4% paraformaldehyde/PBS, mounted with fluorescent mounting medium (DakoCytomation, Glostrup, Denmark) and viewed under confocal microscopy (LSMZ1; Carl Zeiss MicroImaging GmbH, Berlin, Germany) at excitation wavelengths of 488 nm (for EGFP), 458 nm (for ECFP) and 514 nm (for EYFP and Cy3). The immunocytochemistry staining using E3-targeted anti-Na_v_1.5 antibody (Ab) and Cy3-conjugated secondary antibody (Chemicon, Temecula, CA, USA) was performed as described previously (Xu *et al.* 2005). To achieve an optimal signal-to-noise ratio for each fluorescence signal, sequential scanning with the specific wavelength was used.

### Computer simulations

Computational studies attempting to simulate alterations in heart rate using as a basis the biophysical findings used here used an SA node/atrial model established and implemented on previous occasions (Lei *et al.* 2005).

### Statistical analysis

Data are presented as mean ± SEM (number of cells). Differences were evaluated by Student’s *t*-test and a difference was considered significant if *P*<0.05.

## Results

### Clinical phenotypes and genetic analysis

Figure 1a summarizes the pedigree of the affected family. Full medical histories, physical examinations, echocardiographic scanning and ECG recording using at least two 12-lead ECG recordings on two independent occasions, were obtained from each member. Finally, two 24-h Holter-ECG recordings were performed in each of the genetically affected individuals. The proband (subject III-5) was an 8-year-old boy who presented with shortness of breath and exercise intolerance. His resting 12-lead ECG, corroborated by oesophageal ECG recordings, showed a sinus bradycardia with a heart rate of 40–60 beats min^−1^ consistent with SSS, atrial ectopics and widened QRS complexes with a normal QTc (Fig. 1b). His 24 h Holter recordings revealed numerous sinus pauses whose longest duration was 3.2 s (Fig.1c).

Subject II-2, one of the proband’s uncles, similarly had a history of sinus bradycardia with a heart rate of 45–50 beats min^−1^ and suffered multiple episodes of syncope from his twenties. His resting ECG showed an irregular sinus rhythm with long sinus pauses, a just-demonstrable first degree AV block, and ST-T elevation in leads V1 and V2 (Fig. 1d). The father of the proband, subject II-6 was asymptomatic with a heart rate of 60–65 beats min^−1^ but had a resting ECG showing ST-T elevation in leads V1 and V2 (Fig. 1e), features confirmed by four independent repeat ECGs over 3 years. Both subject II-2 and subject II-6 had not undergone any drug challenge tests directed at unmasking BrS (Wilde *et al.* 2002). Finally, the grandmother, subject I-2, was clinically and electrocardiographically normal. The remaining 14 family members investigated were asymptomatic and had normal ECGs. Table 1 compares ECG parameters and clinical features of the mutation carriers and non-carriers within the family concerned, demonstrating significance differences in heart rate, P wave width, PR interval, QRS duration and extent of ST elevation, but not in QTc interval.

**Table 1:** Summary of ECG parameters and clinical information of mutation carriers and non-carriers

	*n*	Age (years)	Gender M : F	Syncope (yes : no)	HR beats min^−1^	P width (ms)	PR interval (ms)	QRS interval (ms)	QTc (ms)	ST elevation (mm)
R878C carriers	4	37 ± 12	3 : 1	1 : 3	61 ± 4	108 ± 5	190 ± 6	103 ± 2.5	373 ± 23	2.1 ± 0.4
Non-carriers	13	30 ± 5	6 : 7	0 : 13	75 ± 3	91 ± 2	140 ± 11	88 ± 1.6	387 ± 7	0.6 ± 0.1
*P*-value		ns	ns	ns	<0.01	<0.01	<0.05	<0.01	ns	<0.0001

ns, not significant.

**Figure 2:**
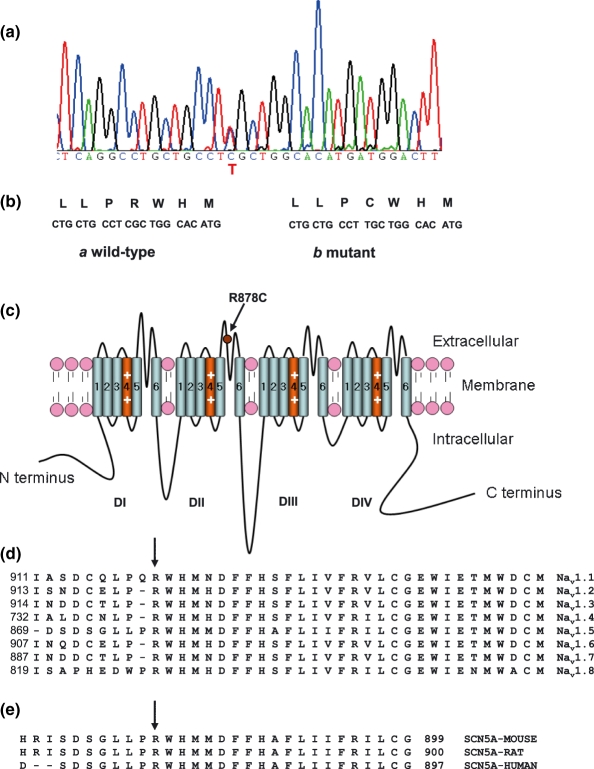
R878C mutation of *SCN5A* sodium channel. (a) Sequencing of SCN5A mutation: heterozygotic mutation C→T transition at nucleotide 2826. (b) Missense mutation of R878C. (c) Position of R878C on Nav1.5 channel. (d–e) Alignment analysis of Na_v_ channel isoforms. R878 is a residue that is highly conserved in Na_v_ channel isoforms (d) and among Scn5a in different rat, mouse and human species (e).

We first screened the proband for *SCN5A* mutations owing to their known association with familial SSS (Lei *et al.* 2007). Having demonstrated genetic alterations in the proband, we then similarly screened the remaining family, with the exceptions of II-9, II-10, III-8 and III-9 who were not available for testing. The same mutation was also screened in 200 controls with normal cardiovascular histories and clinical and ECG examinations and the same ethnic background. DNA sequencing of all the *SCN5A* exons and exon–intron boundaries revealed a novel heterozygous base change in exon 16 (2826 C→T) (Fig. 2a; GenBank accession number: DQ086162) in the proband, leading to the missense mutation in which arginine, R, was replaced by cysteine, C, at position 878 (R878C) (Fig. 2b); this was further verified by SSCP analysis. Amino acid 878 is located at the extracellular linker between transmembrane S5 and S6 in domain II (DII) of the Na_v_1.5 protein (Fig. 2c), which forms part of the channel pore, consistent with an effect of mutation R878C on permeation through Na_v_1.5 channels. Comparison of aligned amino acid sequences that demonstrate that R878 is highly conserved among different human Na_v_ channel isoforms (Fig. 2d). Furthermore, it is also highly conserved among Scn5a in different, rat, mouse and human species (Fig. 2e). We also found R878C in II-2, II-6 and I-2 (Fig. 1a) but not in the remaining members of the family studied. The functional consequences of the R878C Na_v_1.5 were then investigated by biophysical and modelling studies.

### Biophysical characterization

**Figure 3:**
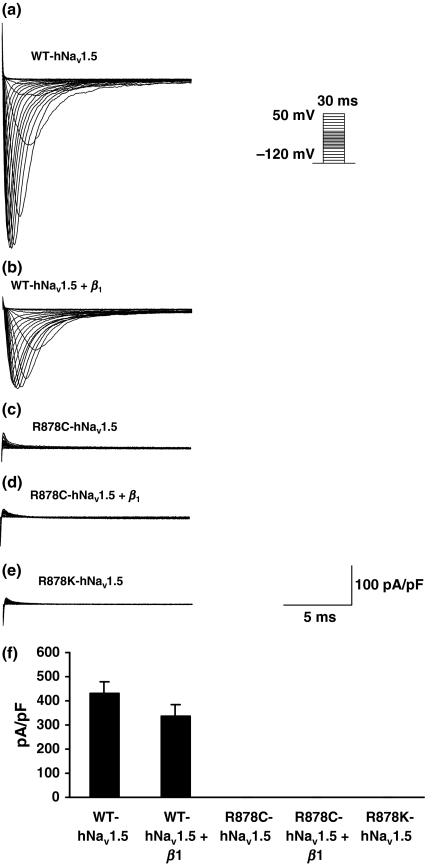
Characterization of *i*_Na_ of WT and mutant channels in HEK293 cells. Inset: HEK293 ells were clamped at a holding potential of −120 mV and subjected to test voltage steps each lasting 30 ms that were made to membrane potentials between −110 and +50 mV in 5 or 10 mV increments and imposed at a pulsing frequency of 1.0 Hz. (a, b) WT-hNa_v_1.5 and WT-hNa_v_1.5 with β_1_ subunit. (c, d) Mutant R878C-hNa_v_1.5 and mutant R878C-hNa_v_1.5 with β_1_ subunit. (e) Substitution of R878K. (f) Bar graph summarizing peak *i*_Na_ (pA pF^−1^) in each of the experimental situations above.

The biophysical studies suggested that the above mutation results in a loss of detectable Na^+^ channel function. Cells were clamped at a holding potential of −120 mV and subjected to 30 ms duration depolarization pulses between −110 and +50 mV in 5 or 10 mV increments at pulse frequency of 1.0 Hz. First, HEK293 cells transiently transfected with (0.5 μg) plasmid DNA for either WT-hNa_v_1.5 or R878C-hNa_v_1.5 were investigated in the whole-cell configuration. Na^+^ currents, *i*_Na_, recorded from the HEK293 cells transfected with plasmid for WT-hNa_v_1.5 showed average peak current densities of 432.2 ± 49.16 pA pF^−1^ (Fig. 3a,f) (*n* = 10 cells). Similar currents were observed at a mean peak level of 334.3 ± 47.56 pA pF^−1^ (*n* = 7 cells) with transfection of half the dose of WT-hNa_v_1.5 plasmid DNA in combination with the same dose of β_1_-subunit plasmid, known to increase hNa_v_1.5 expression (Qu *et al.* 1995, Xiao *et al.* 2000, Herfst *et al.* 2003) and might be involved in the trafficking or membrane insertion of the Na^+^ channels. (Fig. 3b,f). In contrast, cells transfected with R878C-hNa_v_1.5 plasmid DNA (*n* = 20) did not show any detectable *i*_Na_ whether in the absence (Fig. 3c,f) or presence of β_1_-subunit plasmid (Fig. 3d,f). Secondly, similar results were obtained in a second independent expression system produced by injection of R878C-hNa_v_1.5 cRNA into *Xenopus* oocytes studied using a two-microelectrode voltage clamp technique. In these experiments, all the different cRNAs were each diluted to a standard concentration of 0.04 ng μL^−1^. Furthermore, Na^+^ currents were detectable even following injection of WT-hNa_v_1.5 cRNA made to a 50–100-fold dilution. In contrast, Na^+^ currents were undetectable even following injection of R878C-hNa_v_1.5 cRNA prepared undiluted at a 1.5 μg μL^−1^.

### The alterations in Na^+^ channel function critically depend on the R878 residue

These findings thus suggest that substitution of the hydrophilic positively charged arginine with the hydrophobic uncharged amino acid cysteine results in a loss of detectable channel function. However, residue 878 in Na_v_1.5 is located at the extracellular linker between S5 and S6 of domain II (DII), a critical region that forms part of the channel pore. There is therefore the possibility that arginine has a more specific role than simply providing a positive charge influencing the local electrostatic field essential for channel function. Such a notion would be consistent with a comparison of aligned amino acid sequences that demonstrate that R878 is highly conserved among Na_v_ channel isoforms (Fig. 2d,e). This hypothesis was explored by expressing another mutant hNa_v_1.5 construct in which the arginine R878 was replaced by the similarly positively charged lysine (K) to give the substitution R878K, through transient transfection into HEK293 cells. Figure 3e,f shows that such cells also failed to produce any detectable *i*_Na_ (*n* = 9).

### WT-hNa_v_1.5 function persists in the presence of R878C-hNa_v_1.5

The *Xenopus* expression system was then used to quantitatively explore the possible effects of gene dosage and for the presence or absence of any interactions between WT and mutant gene expression. This was achieved by introduction of different combinations of a standard dose of cRNAs encoding each protein variant. The initial experiments explored the effects of identical dosages of cRNA encoding WT-hNa_v_1.5 and mutant R878C-hNa_v_1.5 in different combinations to determine the time course, peak amplitude and voltage dependence of the resulting whole-cell Na^+^ currents.

**Figure 4:**
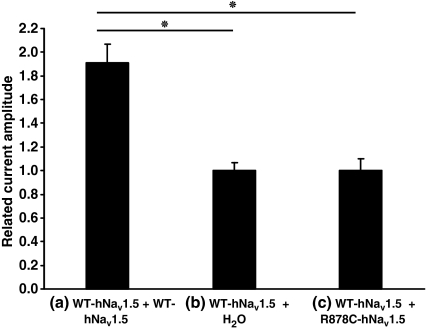
Characterization of peak current amplitudes of WT and mutant channels in *Xenopus* oocytes. Injection of R878C-hNa_v_1.5 cRNA did not alter the current amplitude through WT-hNa_v_1.5 channels (compare WT-hNa_v_1.5 + H_2_O with WT-hNa_v_1.5 + R878C-hNa_v_1.5). In control experiments, we confirmed that a twofold higher WT-hNa_v_1.5 cRNA concentration (WT-hNa_v_1.5 + WT-hNa_v_1.5) resulted in the respective increase in the peak current amplitude. Number of measurements was between 13 and 35, number of oocyte batches was at least 3.

The results of the experiments indicated that the size of *i*_Na_ is simply related to the dose of WT-hNa_v_1.5 cRNA: the mutant R878C-hNa_v_1.5 is non-functional even in the presence of the WT-hNa_v_1.5 channels, and the presence or absence of mutant cRNA neither increases nor decreases the net size of *i*_Na_. Accordingly, the effects of R878C-hNa_v_1.5 were not dependent upon the dominant negative effects of the R878C-hNa_v_1.5 mutation on the function of WT-hNa_v_1.5 on an otherwise normal WT-hNa_v_1.5 expression. First, injections of cRNAs encoding (WT-hNa_v_1.5 + H_2_O) (Fig. 4b) and (WT-hNa_v_1.5 + R878C-hNa_v_1.5) (Fig. 4c) resulted in similar magnitudes of peak *i*_Na_. These were in turn doubled by the doubled dose resulting from injection of cRNA encoding (WT-hNa_v_1.5 + WT-hNa_v_1.5) (Fig. 4a). Secondly, the characteristics of WT-hNa_v_1.5 channels were not affected by co-expression with R878C-hNa_v_1.5. Thus, key parameters describing the voltage dependences of the resulting *i*_Na_ were indistinguishable whether WT hNa_v_1.5 were expressed in the presence or absence of R878C-hNa_v_1.5. Thus the empirical quantification in Table 2 displays statistically identical transition voltages, similar to normal values reported on earlier occasions (Zimmer *et al.* 2002ab,) for inactivation and activation, *V*_h_ and *V*_m_ as well as inactivation time constants τ_h_ at membrane potentials of −35, −25 and −15 mV, and fast, τ_fast_ and slow, τ_slow_, recovery time constants obtained from oocytes injected with WT-hNa_v_1.5 + H_2_O and WT-hNa_v_1.5 plus R878C-hNa_v_1.5 respectively.

**Table 2:** Electrophysiological properties of WT-hNa_v_1.5 channels co-expressed with R878C-hNa_v_1.5 in *Xenopus* oocytes

	Steady-state inactivation		Steady-state activation		Inactivation time constants (τ_h_)				Recovery time constants (τ_rec_)		
Channel	*V*_h_ (mV)	*n*	*V*_m_ (mV)	*n*	−35 mV(ms)	−25 mV(ms)	−15 mV(ms)	*n*	τ_fast_	τ_slow_ (ms)	*n* (ms)
WT-hNa_v_1.5	−64.5 ± 0.4	6	−33.1 ± 1.0	5	3.2 ± 0.7	2.0 ± 0.2	1.5 ± 0.1	5	54.4 ± 0.3	97 ± 18	5
WT-hNa_v_1.5/R878C-hNa_v_1.5	−64.1 ± 0.6	9	−33.2 ± 0.6	9	2.9 ± 0.4	1.7 ± 0.2	1.3 ± 0.1	6	63.6 ± 0.2	105 ± 12	5

Inactivation and recovery curves were fitted mono-exponentially yielding τ_h_ and bi-exponentially yielding τ_fast_ and τ_slow_ respectively. Data are represented as mean ± SEM.

Thirdly, previous papers have reported that incubation with class I anti-arrhythmic agents restored expression of *i*_Na_ in an otherwise functional mutant for I1660V Na^+^ channels that have similarly been related to a Brugada phenotype (Cordeiro *et al.* 2006). Similar procedures also restored K^+^ channel function in similar genetically modified systems (Rajamani *et al.* 2002, Valdivia *et al.* 2004, Anderson *et al.* 2006). However, in the present study, the observed alterations in function persisted in cells treated with class I anti-arrhythmic agents, that would have restored expression in the event of a trafficking phenotype. Such experiments studied HEK293 cells expressing R878C-hNa_v_1.5 that either had been cultured with 200 μm mexiletine for 24 h, or transiently exposed to acute administration of either 200 μm mexiletine or 100 μm lidocaine. In both cases, cells were restored to their normal buffer, with a full washout of any pharmacological agents 30 min before patch-clamp recording, neither of which demonstrated any detectable *i*_Na_ current. Pre-treatment with mexiletine and lidocaine thus failed to result in a recovery of Na^+^ currents in HEK293 cells transfected with R878C -hNa_v_1.5 channels.

### Confocal microscopy results are consistent with persistent surface localization of R878C-hNa_v_1.5 channels

Finally, confocal microscopy results appeared consistent with a persistent surface membrane expression of R878C hNa_v_1.5 channels in intact unpermeabilized cells, consistent with continued trafficking of mutant channels to the plasma membrane, as opposed to a disrupted trafficking of an otherwise functionally normal channel.

**Figure 5:**
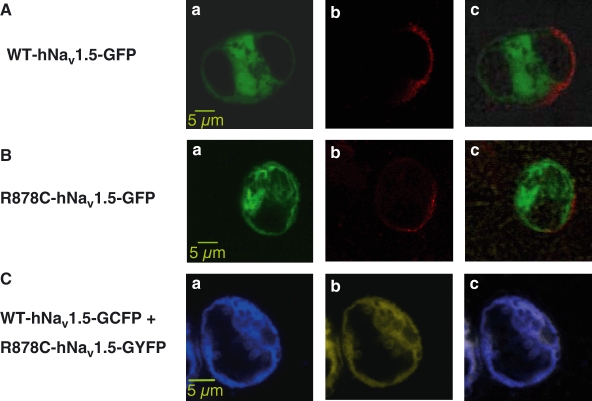
Surface membrane localization of *SCN5A* persists despite the R878C mutation. GFP fluorescence in HEK293 cells of WT-hNa_v_1.5 (Aa) and R878C-hNa_v_1.5 (Ba) and membrane staining of the fluorescent Cy3-conjugated E3-targeted anti-Na_v_1.5 antibody (Ab and Bb) and overlays (Ac and Bc) of these confocal images. Expression and intracellular location following co-transfection with plasmids for pECFP-WT-hNa_v_1.5 and pEYFP-R878C- hNa_v_1.5: exposure to their respective excitation wavelengths confirms co-expression of both WT-hNa_v_1.5-CFP (Ca) and R878C hNa_v_1.5-YFP (Cb) with similar subcellular localizations as reflected in the overlaid images (Cc). Scale bars = 5 μm for all panels.

These studies first transfected HEK293 cells with pEGFP-WT-hNa_v_1.5 and pEGFP-R878C-hNa_v_1.5 constructs respectively. The plasma membrane localization of both WT and mutant channels were then confirmed by extracellular application of a novel E3-targeted anti-Na_v_1.5 antibody (Xu *et al.* 2005) raised against the domain 3 extracellular loop of hNa_v_1.5. Figure 5Aa,Ba confirms successful GFP expression of WT-hNa_v_1.5 (Fig. 5Aa), R878C-hNa_v_1.5 (Fig. 5Ba) and staining of the fluorescent Cy3-conjugated secondary antibody (Fig. 5A,B, panel b) respectively. The latter confirms successful surface membrane expression of WT-hNa_v_1.5 and R878C-hNa_v_1.5. The resulting overlays (Fig. 5Ac,Bc) of these confocal images (Fig. 5Aa and b, and Ba and b) confirm an extracellular location of the expressed protein in both WT-hNa_v_1.5 and R878C-hNa_v_1.5-expressing cells. These results suggest a persistent trafficking and expression of mutant channel R878C-hNa_v_1.5 to the plasma membrane. Secondly, studies were next made of the expression and intracellular location in HEK293 cells co-transfected with plasmids for pECFP-WT-hNa_v_1.5 and pEYFP-R878C-hNa_v_1.5. Exposure to their respective excitation wavelengths confirmed that both WT-hNa_v_1.5-CFP (Fig. 5Ca) and R878C-hNa_v_1.5-YFP (Fig. 5Cb) were co-expressed. Furthermore the overlaid images further demonstrated that the two variants had similar subcellular localizations (Fig. 5Cc). These findings are also consistent with a normal trafficking of the R878C-hNa_v_1.5 channel. Taken together these findings suggest that the loss of Na^+^ current resulting from the R878C mutation reflects loss of function of normally expressed Na^+^ channels rather than altered trafficking of otherwise functional channels.

### Computational analysis of possible functional consequences of the R878C mutation in SAN pacemaker function

**Figure 6:**
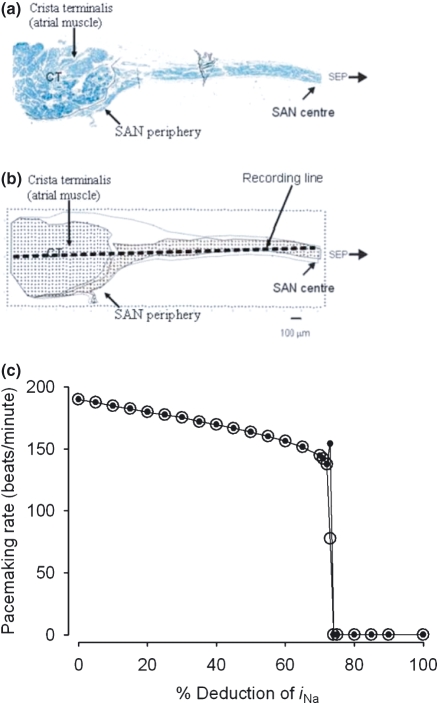
Results of computational modelling of sino-atrial (SA) node function as a result of the R878C mutation. (a) Toluidine blue-stained tissue section through the SA node and its surrounding atrial muscle of the crista terminalis cut through the leading pacemaker site of the rabbit heart. (b) Model of the SA node and its surrounding atrium. (c) Computer simulations of the effect on pacemaker activity by reduction in Na^+^ channel function.

The biophysical studies above suggest that expression systems generated by the introduction of cRNA for R878C-hNa_v_1.5 channel in place of WT-hNa_v_1.5 in *Xenopus* oocytes show a corresponding reduction in the size of Na^+^ current. This would predict that heterozygotes for this mutation show a 50% reduction in expression of functional Na^+^ channels. This possible effect of alterations in *i*_Na_ on pacemaker rate that might thereby explain the clinical phenotypes was assessed in computer simulations based upon an established gradient model of the intact SAN and atrium (Fig. 6a,b) (Zhang *et al.* 2000, Lei *et al.* 2005). The modelling assumed a SAN centre of radius 1.5 mm that contained a uniform density of tetrodotoxin (TTX)-sensitive neuronal Na^+^ channels, and began by assuming that these would give a maximum current of 10 pA pF^−1^. The surrounding SAN periphery was assumed to have an annular radius of 1.5 mm and to contain both neuronal and cardiac Na^+^ channels at a density that increased towards the periphery giving maximum currents from 10 to 60 pA pF^−1^ towards the periphery. Remaining ionic current densities were as reported previously for rabbit SAN (Honjo *et al.* 1996, 1999, Lei *et al.* 2000, 2001). The AP was first initiated in the SAN centre, then allowed to propagate towards its periphery and into the atrial muscle (Lei *et al.* 2007). Numerical simulations were then used to investigate the effect of progressively reducing *i*_Na_ density selectively both in the SAN centre and in the SAN periphery adjacent to the atrium. Figure 6c confirms a continuous decline in the resulting heart rates between 100% and 70% expression of g_Na_ beyond which there was a complete failure of pacemaker activity. Nevertheless, ∼50% reductions in g_Na_ did produce appreciable reductions in heart rate comparable to the clinical observations.

## Discussion

This study describes the physiological correlates of clinical findings within the pedigree of a Chinese family within which a novel mutation (R878C) of the hNa_v_1.5 channel was identified for the first time. Detailed genomic analysis was correlated with results from full clinical examinations of available family members. The same mutation was also screened in 200 controls with normal cardiovascular histories and clinical and ECG examinations and the same ethnic background. DNA sequencing of all the *SCN5A* exons and exon–intron boundaries revealed a novel heterozygous base change in exon 16 (2826 C→T) that would be expected to result in substitution of an arginine in residue 878. This amino acid residue R878 is likely to be critical for Na^+^ channel function. Thus, alignment analysis indicated that R878 is a residue in domain II of S5-S6 that shows a highly conserved arginine residue amongst Na_v_ channel isoforms. Furthermore, R878 is located in the pore-forming region of domain II close to the selectivity filter DEKA and a highly conserved EEDD ring of charge (Xiong *et al.* 2006).

The four family members who proved to be mutation carriers on genetic analysis in the present study showed a range of phenotypic characteristics that variously appeared in childhood (proband) and adult life (uncle of the proband). All of these have nevertheless been associated with cardiac Na^+^ channel mutations on previous occasions. This is in contrast to our recent report of a simpler clinical picture presenting a full and similar penetrance in a family pedigree showing LQT2 (Zhang *et al.* 2007). Thus, a comparison of ECG characteristics suggested a significant slowing of SA, atrioventricular and intraventricular conduction and a significant incidence of sinus bradycardia consistent with SSS and persistent ST elevation in carriers when these were compared with non-carriers from the same family. The present report adds a novel Na^+^ channel variant to the four mutations within the same domain II S5-S6 region, S871fs+9X, F891I, S896S and S910L, that have already been described (Priori *et al.* 2002). All of these were also missense mutations. However, these merely resulted in BrS (Priori *et al.* 2002). Furthermore, earlier studies did not observe the wide range of ECG changes or pursue the fuller cellular functional studies reported here.

These findings nevertheless also add a novel Na^+^ channel variant to available recent reports that have associated *SCN5A* mutations with a range of phenotypes extending even to SSS (Bezzina *et al.* 1999, Veldkamp *et al.* 2000, Benson *et al.* 2003, Groenewegen *et al.* 2003, Makiyama *et al.* 2005, Remme *et al.* 2006). There have been 13 previous *SCN5A* mutations associated with familial SSS by itself or in combination with BrS or LQTS have thus far been identified. Of these, two occurred with a gain of function, five a loss of function and four appeared to result in a total absence of *i*_Na_ in common with the mutation described here (Lei *et al.* 2007). Six heterozygous *SCN5A* mutations have been associated with an autosomal recessive congenital SSS with complete penetrance also associated with conduction disorders including evidence for latent atrioventricular conduction system disease. Two of these mutations produced non-functional Na^+^ channels when expressed in stable mammalian cells (Benson *et al.* 2003). These clinical features correlated with reports from experimental studies in heterozygous *Scn5a*^+/−^mouse models with a targeted heterozygous disruption of *Scn5a* (Papadatos *et al.* 2002, Lei *et al.* 2005). Such reports directly implicated Na_v_1.5 channel currents in arrhythmic phenomena at widespread cardiac sites. These included the conduction of APs through the SAN, from the SAN to atrial muscle, and within the ventricles. They also suggested that these have indirect effects in the maintenance of normal SAN pacemaker rhythm and rate (Lei *et al.* 2005).

The functional consequences of the R878C Na_v_1.5 mutation reported here were then investigated by biophysical and modelling studies. Both HEK293 cells and *Xenopus* oocytes were used as complementary expression systems. Normal Na^+^ currents were observed following expression of WT-hNa_v_1.5 channels in both HEK293 or oocyte cells, the latter even following injection of a 50- to 100-fold dilution of WT-hNa_v_1.5 cRNA. Such *i*_Na_ was observed even with co-expression of half the dose of WT-hNa_v_1.5 cRNA. In contrast, patch-clamp recordings in either HEK293 cells transfected with the construct or voltage-clamped *Xenopus* oocytes injected with cRNA encoding the R878C-hNa_v_1.5 channel failed to show detectable *i*_Na_, even where undiluted cRNA was used for oocyte injection. Furthermore, the presence of such Na^+^ channel function was highly dependent on and specific to the residue R878. Thus, transfection of the hNa_v_1.5 construct, containing a similarly positively charged lysine to give the substitution R878K, into HEK293 cells did not recover Na^+^ channel function. These findings complement previous reports of highly conserved positions that were critical for channel function. Thus insertion of aspartate (1795insD) in the carboxy terminus of SCN5A can result in either BrS or LQTS (Bezzina *et al.* 1999, Veldkamp *et al.* 2000), and mutation of the same residue to a histidine (Y1795H) or cysteine (Y1795C) also results in BrS and LQTS respectively. These previous findings indicate the proximal region of the C-terminus as potentially important in Na^+^ channel function (Rivolta *et al.* 2001). Our study points out that R878 is similarly highly conserved, and that the replacements of either R878C or R878K failed to produce any detectable *i*_Na_ despite both amino acids carrying similar positive charge.

To test for the effect of the simultaneously produced R878C-hNa_v_1.5 mutant on the expression of WT-hNa_v_1.5 *in Xenopus* oocytes, we co-injected equal amounts of the respective cRNAs into oocytes and determined the peak current amplitudes and kinetic parameters of the whole-cell Na^+^ currents. R878C-hNa_v_1.5 did not exert dominant negative phenotypic effects: *i*_Na_ was simply determined by the amount of WT-hNa_v_1.5 cRNA. Doubling the dose of WT-hNa_v_1.5 cRNA doubled *i*_Na_. *i*_Na_ amplitudes and activation and inactivation characteristics were similar with WT-hNa_v_1.5 cRNA given alone or in combination with equal doses of R878C-hNa_v_1.5 cRNA. Furthermore, steady-state activation, steady-state inactivation, inactivation time constants and recovery from inactivation were indistinguishable in WT-hNa_v_1.5 plus R878C-hNa_v_1.5 vs. WT-hNa_v_1.5 channels. These data indicate that the mutant R878C is non-functional also in the presence of WT channels and that WT-hNa_v_1.5 kinetics is not affected.

Additional biophysical and immunochemical experiments provided further evidence that the presence of the mutation probably resulted in a persistent expression of non-functional Na^+^ channels, as opposed to abolishing the trafficking of otherwise functionally normal channels to the cell surface membrane. Thus Na^+^ channel function in HEK293 cells with R878C-hNa_v_1.5 was not restored by transient exposure to either mexiletine (200 μm) or lidocaine (100 μm), manoeuvres that have previously been used to restore channel trafficking (Rajamani *et al.* 2002, Valdivia *et al.* 2004, Anderson *et al.* 2006, Cordeiro *et al.* 2006). In the above respects, our studies complement earlier reports from the four heterozygous *SCN5A* mutations T187I, D356N, K1578fs/52, and R1623X that were similarly linked with BrS, SSS and atrioventricular block mentioned above (Makiyama *et al.* 2005). All these also resulted in non-functional channels, an absence of any dominant negative effects on WT channels and in which *i*_Na_ was also not restored by mexiletine (Makiyama *et al.* 2005), suggesting that the changes may be a general consequence of mutations in *SCN5A.* Furthermore, extracellular application of E3-targeted anti-Na_v_1.5 antibody yielded a significant plasma membrane expression of both WT and mutant channels. This suggests a persistent expression of mutant but non-functional Na^+^ channels despite the complete abolition of Na^+^ currents in the homozygote, consistent with a continued expression of non-functional channels rather than a total failure of expression of otherwise normal channels.

As the affected members in the family concerned were heterozygotes, one would expect that Na^+^ currents in the patients’ cardiomyocytes should be reduced by 50%, i.e. corresponding to the level of WT-hNa_v_1.5 channels produced from the unaffected allele. Numerical modelling demonstrated the effects upon SAN function as reflected in heart rate of a progressive reduction in Na_v_1.5 current resulting from increasing proportions of non-functional mutant channels in an established model of SAN-atrium tissue (Zhang *et al.* 2000, Lei *et al.* 2005). This produced graded reductions in heart rate at least over the reduction region of *i*_Na_ as seen in clinical cases, whose physiological correlates are examined here.

In conclusion, we have identified and characterized a novel mutation at a highly conserved pore residue in domain II S5-S6 of the cardiac type Na^+^ channel in a three-generation Chinese family that complements previous studies reporting a range of, rather than a single clinical, phenotype.

## References

[b1] Akai J, Makita N, Sakurada H, Shirai N, Ueda K, Kitabatake A, Nakazawa K, Kimura A, Hiraoka M (2000). A novel SCN5A mutation associated with idiopathic ventricular fibrillation without typical ECG findings of Brugada syndrome. FEBS Lett.

[b2] Anderson CL, Delisle BP, Anson BD, Kilby JA, Will ML, Tester DJ, Gong Q, Zhou Z, Ackerman MJ, January CT (2006). Most LQT2 mutations reduce Kv11.1 (hERG) current by a class 2 (trafficking-deficient) mechanism. Circulation.

[b3] Bennett PB, Yazawa K, Makita N, George AL (1995). Molecular mechanism for an inherited cardiac arrhythmia. Nature.

[b4] Benson DW, Wang DW, Dyment M, Knilans TK, Fish FA, Strieper MJ, Rhodes TH, George AL (2003). Congenital sick sinus syndrome caused by recessive mutations in the cardiac sodium channel gene (SCN5A). J Clin Invest.

[b5] van den Berg MP, Wilde AA, Viersma TJW, Brouwer J, Haaksma J, van der Hout AH, Stolte-Dijkstra I, Bezzina TCR, Van Langen IM, Beaufort-Krol GC, Cornel JH, Crijns HJ (2001). Possible bradycardic mode of death and successful pacemaker treatment in a large family with features of long QT syndrome type 3 and Brugada syndrome. J Cardiovasc Electrophysiol.

[b6] Bezzina C, Veldkamp MW, van Den Berg MP, Postma AV, Rook MB, Viersma JW, van Langen IM, Tan-Sindhunata G, Bink-Boelkens MT, van Der Hout AH, Mannens MM, Wilde AA (1999). A single Na(+) channel mutation causing both long-QT and Brugada syndromes. Circ Res.

[b7] Chen Q, Kirsch GE, Zhang D, Brugada R, Brugada J, Brugada P, Potenza D, Moya A, Borggrefe M, Breithardt G (1998). Genetic basis and molecular mechanism for idiopathic ventricular fibrillation. Nature.

[b8] Cordeiro JM, Barajas-Martinez H, Hong K, Burashnikov E, Pfeiffer R, Orsino AM, Wu YS, Hu D, Brugada J, Brugada P, Antzelevitch C, Dumaine R, Brugada R (2006). Compound heterozygous mutations P336L and I1660V in the human cardiac sodium channel associated with the Brugada syndrome. Circulation.

[b9] Groenewegen WA, Firouzi M, Bezzina CR, Vliex S, van Langen IM, Sandkuijl L, Smits JP, Hulsbeek M, Rook MB, Jongsma HJ, Wilde AA (2003). A cardiac sodium channel mutation cosegregates with a rare connexin40 genotype in familial atrial standstill. Circ Res.

[b10] Herfst LJ, Potet F, Bezzina CR, Groenewegen WA, Le Marec H, Hoorntje TM, Demolombe S, Baro I, Escande D, Jongsma HJ, Wilde AA, Rook MB (2003). Na^+^ channel mutation leading to loss of function and non-progressive cardiac conduction defects. J Mol Cell Cardiol.

[b11] Honjo H, Boyett MR, Kodama I, Toyama J (1996). Correlation between electrical activity and the size of rabbit sino-atrial node cells. J Physiol.

[b12] Honjo H, Lei M, Boyett MR, Kodama I (1999). Heterogeneity of 4-aminopyridine-sensitive current in rabbit sinoatrial node cells. Am J Physiol.

[b13] Lei M, Honjo H, Kodama I, Boyett MR (2000). Characterisation of the transient outward K^+^ current in rabbit sinoatrial node cells. Cardiovasc Res.

[b14] Lei M, Honjo H, Kodama I, Boyett MR (2001). Heterogeneous expression of the delayed-rectifier K^+^ currents i(K,r) and i(K,s) in rabbit sinoatrial node cells. J Physiol.

[b15] Lei M, Goddard C, Liu J, Leoni AL, Royer A, Fung SS, Xiao G, Ma A, Zhang H, Charpentier F, Vandenberg JI, Colledge WH, Grace AA, Huang CL (2005). Sinus node dysfunction following targeted disruption of the murine cardiac sodium channel gene Scn5a. J Physiol.

[b16] Lei M, Zhang H, Grace AA, Huang CL (2007). SCN5A and sinoatrial node pacemaker function. Cardiovasc Res.

[b17] Makiyama T, Akao M, Tsuji K, Doi T, Ohno S, Takenaka K, Kobori A, Ninomiya T, Yoshida H, Takano M (2005). High risk for bradyarrhythmic complications in patients with Brugada syndrome caused by SCN5A gene mutations. J Am Coll Cardiol.

[b18] Papadatos GA, Wallerstein PM, Head CE, Ratcliff R, Brady PA, Benndorf K, Saumarez RC, Trezise AE, Huang CL, Vandenberg JI, Colledge WH, Grace AA (2002). Slowed conduction and ventricular tachycardia after targeted disruption of the cardiac sodium channel gene Scn5a. Proc Natl Acad Sci USA.

[b19] Priori SG, Napolitano C, Gasparini M, Pappone C, Della Bella P, Giordano U, Bloise R, Giustetto C, De Nardis R, Grillo M, Ronchetti E, Faggiano G, Nastoli J (2002). Natural history of Brugada syndrome: insights for risk stratification and management. Circulation.

[b20] Qu Y, Isom LL, Westenbroek RE, Rogers JC, Tanada TN, McCormick KA, Scheuer T, Catterall WA (1995). Modulation of cardiac Na^+^ channel expression in Xenopus oocytes by beta 1 subunits. J Biol Chem.

[b21] Rajamani S, Anderson CL, Anson BD, January CT (2002). Pharmacological rescue of human K(+) channel long-QT2 mutations: human ether-a-go-go-related gene rescue without block. Circulation.

[b22] Remme CA, Verkerk AO, Nuyens D, van Ginneken AC, van Brunschot S, Belterman CN, Wilders R, van Roon MA, Tan HL, Wilde AA, Carmeliet P, de Bakker JM, Veldkamp MW, Bezzina CR (2006). Overlap syndrome of cardiac sodium channel disease in mice carrying the equivalent mutation of human SCN5A-1795insD. Circulation.

[b23] Rivolta I, Abriel H, Tateyama M, Liu H, Memmi M, Vardas P, Napolitano C, Priori SG, Kass RS (2001). Inherited Brugada and long QT-3 syndrome mutations of a single residue of the cardiac sodium channel confer distinct channel and clinical phenotypes. J Biol Chem.

[b24] Smits JP, Koopmann TT, Wilders R, Veldkamp MW, Opthof T, Bhuiyan ZA, Mannens MM, Balser JR, Tan HL, Bezzina CR, Wilde AA (2005). A mutation in the human cardiac sodium channel (E161K) contributes to sick sinus syndrome, conduction disease and Brugada syndrome in two families. J Mol Cell Cardiol.

[b25] Syrris P, Murray A, Carter ND, McKenna WM, Jeffery S (2001). Mutation detection in long QT syndrome: a comprehensive set of primers and PCR conditions. J Med Genet.

[b26] Tan HL, Bezzina CR, Smits JP, Verkerk AO, Wilde AA (2003). Genetic control of sodium channel function. Cardiovasc Res.

[b27] Valdivia CR, Tester DJ, Rok BA, Porter CB, Munger TM, Jahangir A, Makielski JC, Ackerman MJ (2004). A trafficking defective, Brugada syndrome-causing SCN5A mutation rescued by drugs. Cardiovasc Res.

[b28] Veldkamp MW, Viswanathan PC, Bezzina C, Baartscheer A, Wilde AA, Balser JR (2000). Two distinct congenital arrhythmias evoked by a multidysfunctional Na(+) channel. Circ Res.

[b29] Wang Q, Shen J, Splawski I, Atkinson D, Li Z, Robinson JL, Moss AJ, Towbin JA, Keating MT (1995). SCN5A mutations associated with an inherited cardiac arrhythmia, long QT syndrome. Cell.

[b30] Wang T, Waters CT, Rothman AM, Jakins TJ, Romisch K, Trump D (2002). Intracellular retention of mutant retinoschisin is the pathological mechanism underlying X-linked retinoschisis. Hum Mol Genet.

[b31] Wang DW, Desai RR, Crotti L, Arnestad M, Insolia R, Pedrazzini M, Ferrandi C, Vege A, Rognum T, Schwartz PJ, George AL (2007). Cardiac sodium channel dysfunction in sudden infant death syndrome. Circulation.

[b32] Wilde AA, Antzelevitch C, Borggrefe M, Brugada J, Brugada R, Brugada P, Corrado D, Hauer RN, Kass RS, Nademanee K, Priori SG, Towbin JA (2002). Proposed diagnostic criteria for the Brugada syndrome. Eur Heart J.

[b33] Xiao YF, Wright SN, Wang GK, Morgan JP, Leaf A (2000). Coexpression with beta(1)-subunit modifies the kinetics and fatty acid block of hH1(alpha) Na(+) channels. Am J Physiol Heart Circ Physiol.

[b34] Xiong W, Farukhi YZ, Tian Y, Disilvestre D, Li RA, Tomaselli GF (2006). A conserved ring of charge in mammalian Na^+^ channels: a molecular regulator of the outer pore conformation during slow inactivation. J Physiol.

[b35] Xu SZ, Zeng F, Lei M, Li J, Gao B, Xiong C, Sivaprasadarao A, Beech DJ (2005). Generation of functional ion-channel tools by E3 targeting. Nat Biotechnol.

[b36] Zhang H, Holden AV, Kodama I, Honjo H, Lei M, Varghese T, Boyett MR (2000). Mathematical models of action potentials in the periphery and center of the rabbit sinoatrial node. Am J Physiol Heart Circ Physiol.

[b37] Zhang Y, Zhou N, Jiang W, Peng J, Wan H, Huang C, Xie Z, Huang CL, Grace AA, Ma A (2007). A missense mutation (G604S) in the S5/pore region of HERG causes long QT syndrome in a Chinese family with a high incidence of sudden unexpected death. Eur J Pediatr.

[b38] Zimmer T, Biskup C, Bollensdorff C, Benndorf K (2002a). The beta1 subunit but not the beta2 subunit colocalizes with the human heart Na^+^ channel (hH1) already within the endoplasmic reticulum. J Membr Biol.

[b39] Zimmer T, Biskup C, Dugarmaa S, Vogel F, Steinbis M, Bohle T, Wu YS, Dumaine R, Benndorf K (2002b). Functional expression of GFP-linked human heart sodium channel (hH1) and subcellular localization of the a subunit in HEK293 cells and dog cardiac myocytes. J Membr Biol.

